# Unveiling tumor senescence-driven prognostic heterogeneity via MALISS in stage II/III colorectal cancer

**DOI:** 10.3389/fimmu.2025.1744719

**Published:** 2026-01-06

**Authors:** Xinyu Liu, Bingyao Liu, Yuhao Tong, Xingyu Zhu, Yaodong Sang, Feng Gao, Xiangyun Niu, Youyong Tang, Kang Xu, Hao Chen, Wei Chong, Leping Li

**Affiliations:** 1 Department of Gastrointestinal Surgery, Shandong Provincial Hospital Affiliated to Shandong First Medical University, Jinan, China; 2 Shandong Provincial Laboratory of Translational Medicine Engineering for Digestive Tumors, Shandong Provincial Hospital, Jinan, China; 3 Medical Science and Technology Innovation Center, Shandong First Medical University & Shandong Academy of Medical Sciences, Jinan, China; 4 School of Life Science and Technology, Shandong Second Medical University, Weifang, Shandong, China; 5 Institute of Brain Science and Brain-inspired Research, Shandong First Medical University & Shandong Academy of Medical Sciences, Jinan, Shandong, China; 6 Department of Clinical Laboratory, Shandong Provincial Hospital Affiliated to Shandong First Medical University, Jinan, Shandong, China; 7 Clinical Research Center of Shandong University, Clinical Epidemiology Unit Qilu Hospital of Shandong University, Jinan, Shandong, China

**Keywords:** immunosenescence, machine learning, NR1D2, stage II/III colorectal cancer, tumor microenvironment

## Abstract

**Background:**

Prognostic heterogeneity in stage II/III colorectal cancer (CRC) challenges clinical management, yet effective prognostic stratification is still lacking. To address this, we developed a novel machine learning-based signature focused on immunosenescence.

**Methods:**

This study developed a machine learning-based immunosenescence signature (MALISS) using transcriptomic data from 1296 patients. The final 30-gene model was derived via a CoxBoost-Lasso algorithm and validated across multiple independent cohorts.

**Results:**

The MALISS signature effectively stratified patients into high- and low-risk groups with distinct progression-free survival. Functional analysis identified NR1D2 as a key gene promoting tumor migration through cellular senescence. The high-risk group was characterized by a unique mutational landscape, an altered tumor microenvironment, and differential drug sensitivity. Furthermore, a prognostic nomogram integrating MALISS with clinical biomarkers demonstrated improved predictive performance.

**Conclusion:**

MALISS serves as a robust tool for risk stratification and provides valuable insights into tumor biology, offering a promising approach to address prognostic heterogeneity in stage II/III CRC.

## 1 Introduction

According to the latest surveillance data from the International Agency for Research on Cancer (IARC), colorectal cancer (CRC) now ranks third in annual incidence and is the second leading cause of cancer-related deaths worldwide (1). Despite improvements in clinical cure rates through advances in combination therapies such as targeted and immunotherapeutic approaches, 10%–20% of stage II and 30%–40% of stage III CRC patients still face the risk of recurrence (2). Moreover, the prognosis of stage II/III CRC patients is highly heterogeneous, and effective prognostic stratification remains lacking in clinical practice, hindering personalized treatment strategies (3). Therefore, developing a novel prognostic model based on clinical characteristics of stage II/III CRC patients represents a critical clinical unmet need.

Several studies have developed prognostic molecular models for CRC, including those based on lncRNA, metabolism, and immune-related signatures (4–7). However, there remains a lack of emphasis on the comprehensive validation and exploration of various modeling algorithms. Cellular senescence is a stable state of terminal cell cycle arrest. While it was initially considered a barrier to tumorigenesis, a growing body of research now suggests that senescence may enable cells to acquire tumor-promoting properties (8). Meanwhile, we have further refined the combinations of modeling algorithms and signature validation to enhance the model’s credibility. With the advancement of sequencing technologies, genomic, metabolomic, and transcriptomic data are increasingly being integrated with machine learning to predict prognosis and patient survival (9). Clinicians and researchers are now turning to machine learning techniques—such as neural networks, ensemble methods—for patient outcome classification and prediction.

Therefore, this study aimed to identify clinically relevant immunosenescence-related genes in stage II/III CRC patients using a 10-fold or bootstrap machine learning framework. A machine learning-based immunosenescence gene prognostic signature (MALISS) model was developed and validated across four additional independent datasets. Multi-omics approaches and *in vitro* experiments were further employed to explore the biological activities, molecular mechanisms, tumor mutational burden, tumor microenvironment remodeling, and chemotherapy response across different MALISS subgroups. Finally, we developed and validated a nomogram integrating MALISS with clinical and molecular characteristics.

## 2 Material And methods

### 2.1 Study design and public data preprocessing for MALISS model establishment

Gene expression profiles and corresponding clinical features for CRC samples were obtained from datasets in the NCBI GEO and TCGA databases. Additionally, transcriptomic data and drug sensitivity information for CRC cell lines were acquired from the DepMap portal, while proteomic data for colon cancer tissues were derived from the CPTAC dataset.

A total of 1296 stage II/III CRC samples from eight independent datasets, assayed by either RNA-seq or microarray, were selected for prediction model. A large retrospective follow-up dataset comprising GSE14333, GSE37892, GSE39582, and TCGA-CRC was used as the training dataset for model generation. Four independent datasets(GSE12945, GSE143985, GSE103479, and MSK-READ) served as validation datasets for model performance evaluation.

Data refinement involved the exclusion of samples with zero survival time, patients who had undergone preoperative chemotherapy and/or radiotherapy, and cases with missing data regarding recurrence prognosis, staging, or transcriptome. Subsequently, the samples were restricted to stage II/III colorectal cancer patients, and survival time was converted to months to ensure homogeneity. A training dataset of 1000 samples was established for prognostic model development. Model effectiveness was assessed using four independent test datasets (n = 296).

### 2.2 Immunosenescence-related gene signature establishment

In this study, the outcome variables comprised recurrence-specific survival information, defined as the time from surgery to either disease recurrence or the last follow-up, along with recurrence status. The predictors in the signature were the expression levels of immunosenescence genes that had been rigorously screened. To minimize noise from low-expressed or invariant genes, transcriptomic data in the training set were filtered to retain approximately 6000 genes exhibiting the highest median absolute deviation (MAD) (10). These genes were detectable by at least one probeset across all datasets, and for each gene, the probeset with the largest MAD was selected to represent its expression. From the KEGG and ImmuPortdatabases, theimmunosenescence-related pathways were respectively retrieved, involving 2465 genes (11)(**Supporting Information Table S1**). A total of 2465 overlapping genes from these pathways were carried forward for analysis. Univariate Cox regression was performed on these genes, leading to the identification of 177 immunosenescence-related prognostic genes, which were subsequently included in the machine learning framework (**Supplementary Table S2**).

The modeling framework integrated ten distinct machine learning algorithms and their combinations. These methods consisted of Random Survival Forest (RSF), Elastic Net (Enet), Lasso, Ridge, Stepwise Cox, CoxBoost, Partial Least Squares regression for Cox (plsRcox), Supervised Principal Components (SuperPC), Generalized Boosted Regression Modeling (GBM), and Survival Support Vector Machine (survival-SVM). Among these, six algorithms—RSF, Enet, Lasso, Ridge, Stepwise Cox, and CoxBoost—were capable of performing feature selection. A total of 83 modeling strategies, constructed through 10−fold cross−validation or bootstrap resampling, were evaluated to identify the optimal predictive model. Detailed implementation of the algorithm framework and corresponding hyperparameter optimization settings are provided in **Supplementary Table S3**. The model demonstrating the highest average C-index across all validation datasets was selected as the final signature.

### 2.3 Development And validation of the nomogram

A prognostic nomogram for predicting recurrence in stage II/III colorectal cancer (CRC) patients was developed using the ‘rms’ package. Current National Comprehensive Cancer Network (2021) guidelines recommend seven key biomarkers—KRAS, NRAS, BRAF, microsatellite instability (MSI), mismatch repair (MMR), ERBB2 amplification, and NTRK fusion—to guide clinical decision-making (12). Three of these biomarkers were available across both the TCGA and validation datasets (GSE92921 and GSE143985). Using Cox proportional hazards regression, we integrated these molecular features with our previously established gene signature to build a comprehensive prognostic model, which was then translated into a clinically useful nomogram. Decision curve analysis (DCA) was applied to evaluate the clinical net benefit of different models against the “treat-all” and “treat-none” strategies. The predictive performance of the integrated model was further assessed using time-dependent AUC and visualized through restricted mean survival (RMS) curves.

### 2.4 Statistical analysis

All data processing, statistical analyses, and visualization were conducted using R software (version 4.2.2). Associations between continuous variables were assessed using Spearman correlation analysis. Group comparisons for quantitative data were performed with the Wilcoxon rank-sum test or T test for two groups, and analysis of variance (ANOVA) or the Kruskal–Wallis test for multiple groups. Categorical variables were analyzed using two-sided Fisher’s exact test. Survival analyses, including Cox proportional hazards regression and Kaplan–Meier estimation, were carried out with the “survival” package. The predictive accuracy of the MALISS signature for prognosis was evaluated using time-dependent receiver operating characteristic (ROC) curves, with area under the curve (AUC) values computed via the “timeROC” package. Comparisons of prognostic performance between clinical and molecular features and risk scores were implemented using the “compareC” package. All statistical tests were two-sided, and a P value below 0.05 was considered statistically significant. Error bars in figures represent 95% confidence intervals.

### 2.5 Signature characterization

For the characterization of the molecular signature, RNA-sequencing data from TCGA-CRC patients were obtained from the UCSC Xena database (https://xenabrowser.net/datapages/). These data were processed by conversion to TPM format followed by log2 transformation. During both model training and implementation, all gene expression values were standardized by converting them to Z-scores across all samples.

### 2.6 Cell culture and transfection

The HCT15 cell line (RRID: CVCL_0292), which was cultured in RPMI-1640 (Gibco) medium with 10% fetal bovine serum (Vazyme), penicillin-streptomycin (100 U/mL, NCM), was procured from ATCC. The cell was cultured in a 95% air and 5% CO2 environment at 37 °C.

Small interfering RNAs (siRNA) of NR1D2 and negative control (NC) siRNAs were designed and chemically synthesized by Keyybio (Shandong, China). NR1D2 overexpressing plasmid pCMV-EGFP-NR1D2(human)-Neo was purchased from Miaolingbio (Hubei, China). Transfections of siRNA were performed using Lipofectamine2000 Reagent(Thermo Fisher) and plasmids were performed using Lipofectamine3000 Reagent(Thermo Fisher).

### 2.7 Western blot

The transfected cells were lysed by RIPA(Solarbio) with 1% PMSF(Solarbio) to extract the protein. The protein concentration was measured by BCA protein assay kit (Solarbio). After obtaining the OD value, the final concentration of each sample was calculated according to the concentration formula of standard curve calculation. After loading samples, the protein was separated with 10% SDS-PAGE, and then transferred onto PVDF membranes. 5% skimmed milk was used to block the membranes for 1h with 50 rpm decolorization shaker. After washing the membranes with TBS‐t solution, the primary antibodies targeting NR1D2(Proteintech; 13906‐1‐AP), Beta Actin(Proteintech; 66009-1-Ig), P16(Proteintech; 10883‐1‐AP), P21(Cell Signaling Technology; 13426‐1‐AP; 70 kDa), DLST (CST; 5556S; 50 kDa), PDHA1 (Proteintech; 2947) were added for incubation with 50 rpm decolorization at 4 °C for 14h. The secondary antibody(Proteintech) was incubated at 50 rpm, room temperature for 1 h. ECL Kit (Solarbio) was used for detection, and relative quantitative values of the bands were measured by ImageJ software (v.1.4.3.67).

### 2.8 Real-time quantitative PCR

Total RNA was isolated with RNA-easy Isolation Reagent(Vazyme) and reverse transcribed into cDNA by HiScripIII RT SuperMix(Vazyme). We used an Applied Biosystems QuantStudio 1 Real Time PCR system (Applied Biosystems, ThermoFish) to perform quantitative real-time polymerase chain reaction (qRT-PCR) with ChamQ Universal SYBR qPCR Master Mix(Vazyme). The relative expression levels of mRNA were calculated by using the 2−ΔΔCt method, and higher 2−ΔΔCt reflects higher expression.

### 2.9 Transwell assay and wound healing assay

3.5×10^5 HCT15 cells (200 µL of serum-free medium) were seeded onto 8-mm Pore Transwell Inserts(Corning) and 600 µL 10%FBS complete culture medium to the lower compartment for migration assay. After incubation at 37°Cfor 24h, cells on the Transwell Inserts were then fixed with 4% paraformaldehyde for 30 min and stained with hematoxylin solution for 30 min.

For wound healing assay, transfected HCT15 cells were seeded into 6-well plate and used the 200 µL tip to make a vertical line scratch. After washing with PBS, and the serum-free medium was replaced to continue the culture, and the cells were photographed through a microscope at 0 h, 24 h, respectively, and the mobility was measured and calculated by using ImageJ software (v.1.4.3.67).

### 2.10 SA-β-gal staining assay

SA-β-gal staining was performed to assess cellular senescence in HCT15 cells following siRNA transfection. Cells were fixed with 4% paraformaldehyde (PFA) and stained using the Cell Senescence SA-β-Gal Staining Kit (Beyotime).

## 3 Results

### 3.1 Construction of a machine learning-based immunosenescence-related gene signature model

**Figure 1:**
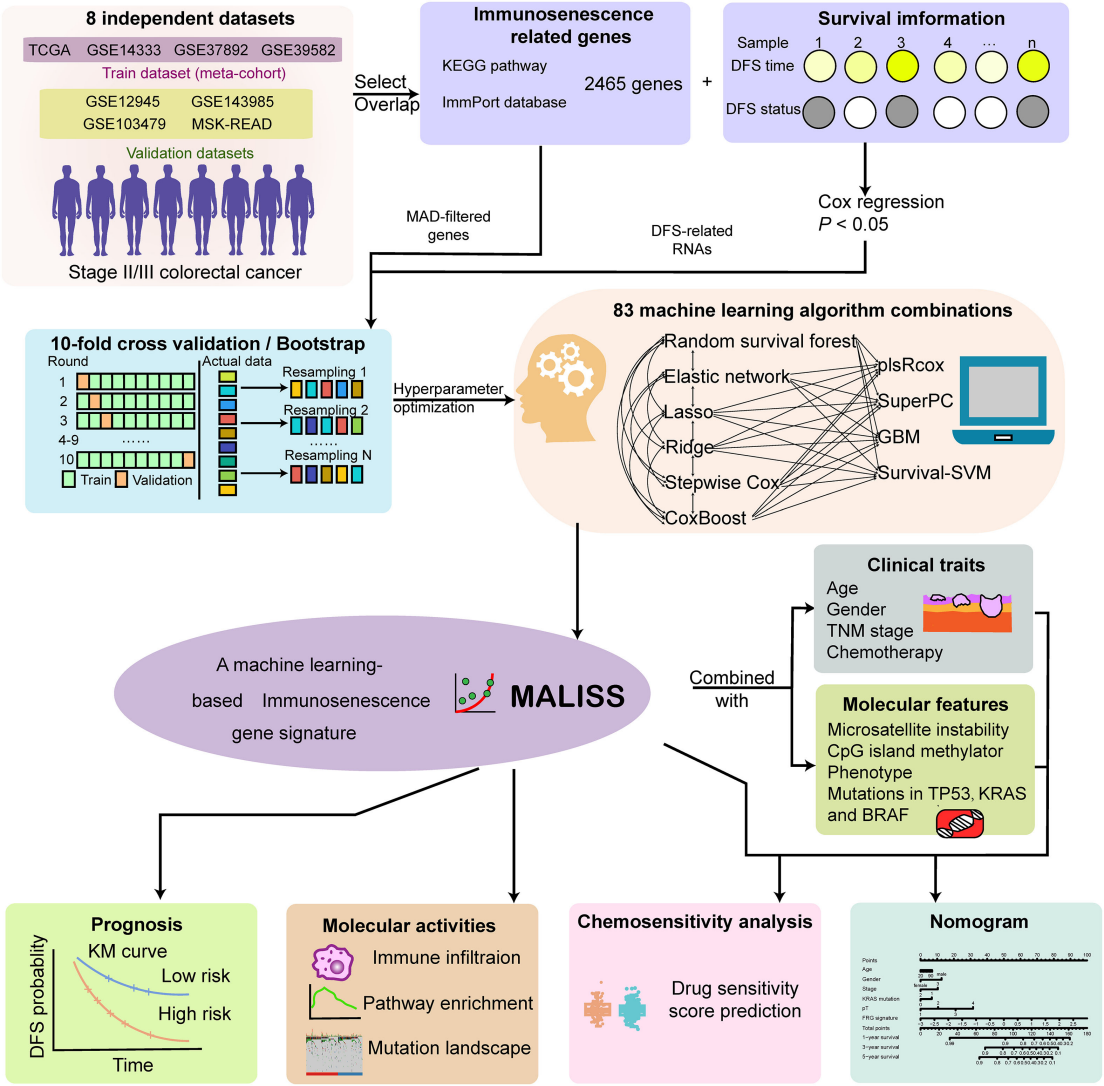
Overall workflow of the study.

To construct a immunosenescence-related gene signature predictive of prognosis in stage II/III colorectal cancer (CRC), we integrated multiple independent datasets. A meta-cohort consisting of TCGA, GSE14333, GSE37892, and GSE39582 was used as the training set, while GSE12945, GSE143985, GSE103479, and MSK-READ served as external validation datasets. We initially selected 2465 immunosenescence-related genes from the KEGG pathway and ImmPort database. Combining with disease-free survival (DFS) time and status information, we performed univariate Cox regression analysis (p < 0.05) to identified 177 immunosenescence-related genes in the training meta-cohort. Subsequently, after performing hyperparameter optimization on the 83 validated predictive models and determining the final combination model, we analyzed the immunosenescence gene signature. Finally, the immunosenescence gene signature effectively stratified patients into distinct prognostic groups and was further analyzed its associated molecular activities. Moreover, integrated with clinical traits and molecular features, it was used to predict drug sensitivity and was incorporated into a nomogram for potential clinical application. (**Figure 1**)

**Figure 2:**
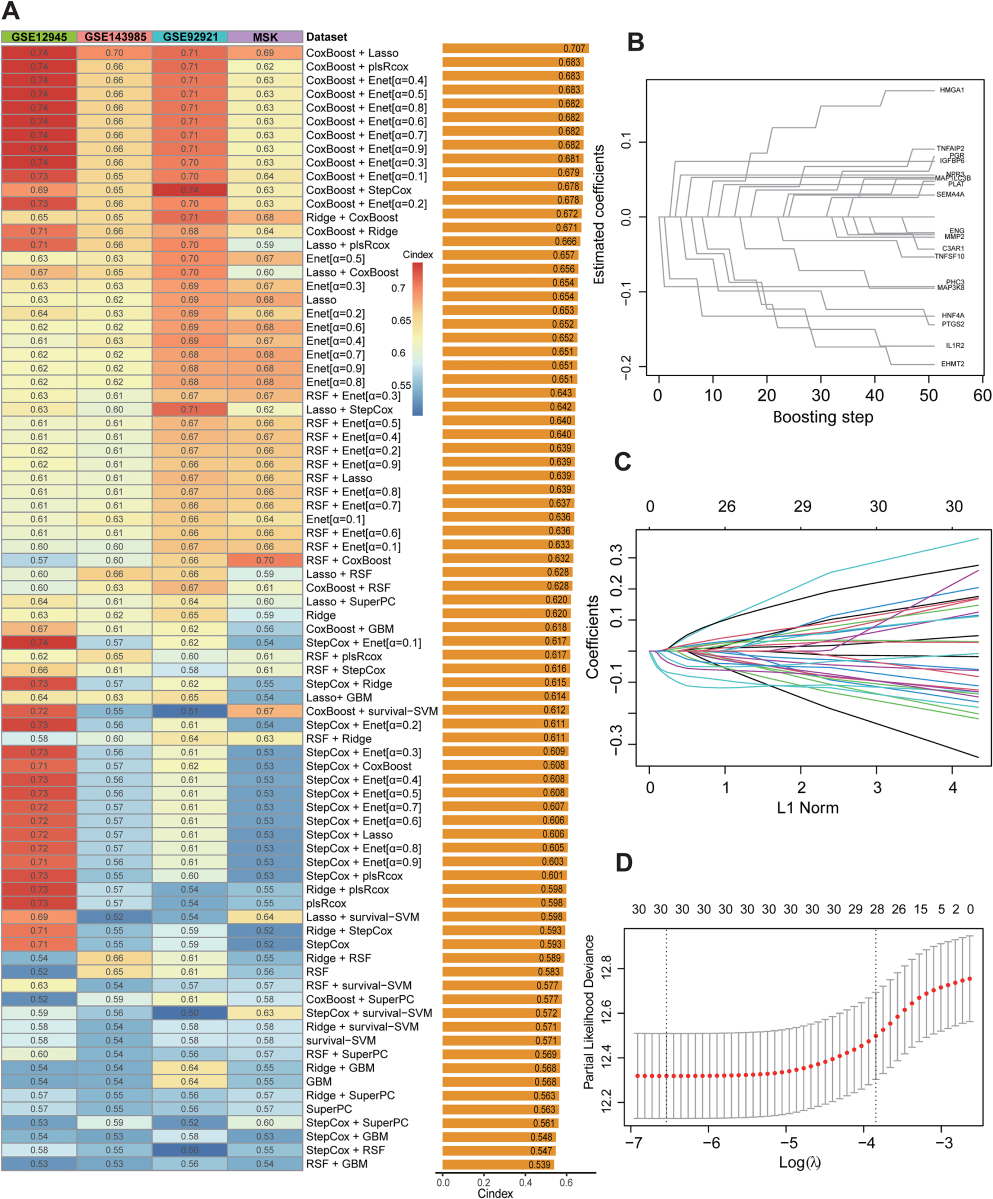
The identification of the best performance signature. **(A)** C-indices of 83 combinations of machine learning prediction models in four independent validation cohorts. **(B)** The coefficient paths of selected immunosenescence-related genes across boosting steps in the CoxBoost model. **(C)** LASSO Coefficient Path Plot. **(D)** LASSO Cross-Validation Error Plot.

Through ten-fold cross-validation or bootstrap resampling of 83 prediction models integrating 10 machine learning algorithms, the CoxBoost-Lasso combination model demonstrated the highest and most robust C-index on the 4 validation datasets (**Figure 2A**). The CoxBoost algorithm was used to preliminarily screen for important predictors iteratively (**Figure 2B**). A total of 31 genes were selected in the CoxBoost algorithm (**Supplementary Table S4**). The LASSO model was applied to optimize the λ value and define the final variable composition of the model (**Figures 2C, D**). Ultimately, we selected the expression of 30 gene features to construct a MAchine Learning-based ImmunoSenescence-related gene Signature(MALISS) model (**Supplementary Table S5**).

### 3.2 Evaluation of MALISS model performance and prognostic value

**Figure 3:**
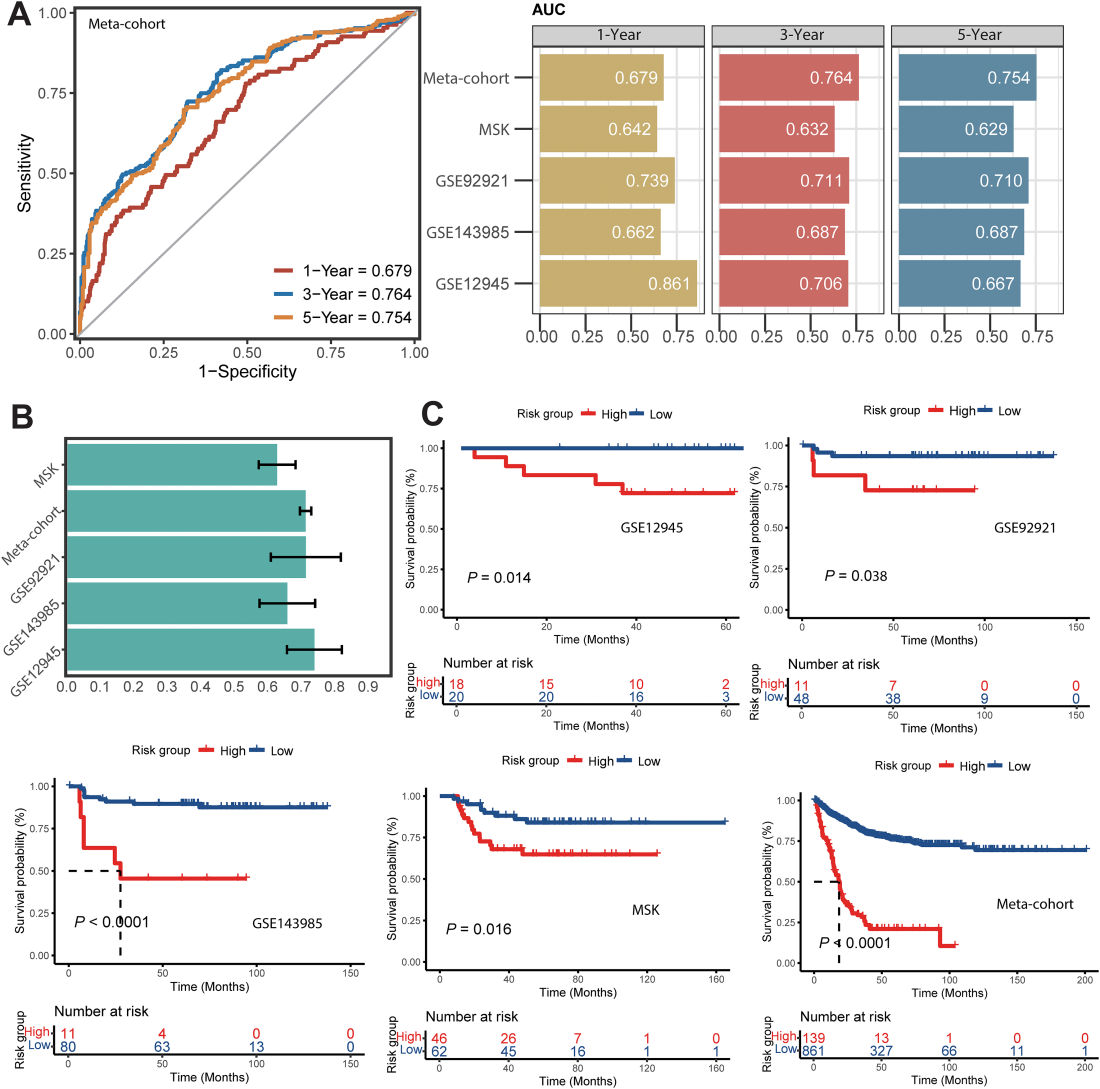
Evaluation of MALISS model performance. **(A)** Time-dependent ROC analysis for predicting 1-, 3-, and 5-year PFS across the training meta-cohort and four validation datasets. **(B)** C-indices of the training dataset and four validation datasets. **(C)** Kaplan–Meier survival curve of PFS with high- and low- score groups of MALISS in the training meta-cohort and four validation datasets.

ROC analysis demonstrated that the MALISS model exhibited a strong discriminative ability for PFS. In the training dataset, it achieved favorable AUC values, with an average of 0.732 (1-year AUC: 0.679; 3-year AUC: 0.764; 5-year AUC: 0.754). The model also showed consistently excellent performance across four independent validation datasets, attaining an average 3-year AUC of 0.684 (**Figure 3A**). Favorable C-index performance was observed in the training dataset and across all validation datasets (**Figure 3B**). Based on the median MALISS score, patients in each dataset were stratified into high-risk and low-risk groups. In both the training dataset and the GSE143985 validation dataset, significant differences in PFS time were observed between the two groups, with all P-values less than 0.0001. In the other three validation datasets, the high-risk group also exhibited significantly shorter PFS time than the low-risk group, with all P-values below 0.05. These results demonstrate that the MALISS model effectively predicts prognosis in CRC patients. Thus, we consider the MALISS model to be a promising biomarker of PFS risk in stage II/III CRC patients.

### 3.3 NR1D2 promotes CRC migration by inducing CRC cell senescence and enhancing the secretion of SASP-associated factors.

**Figure 4:**
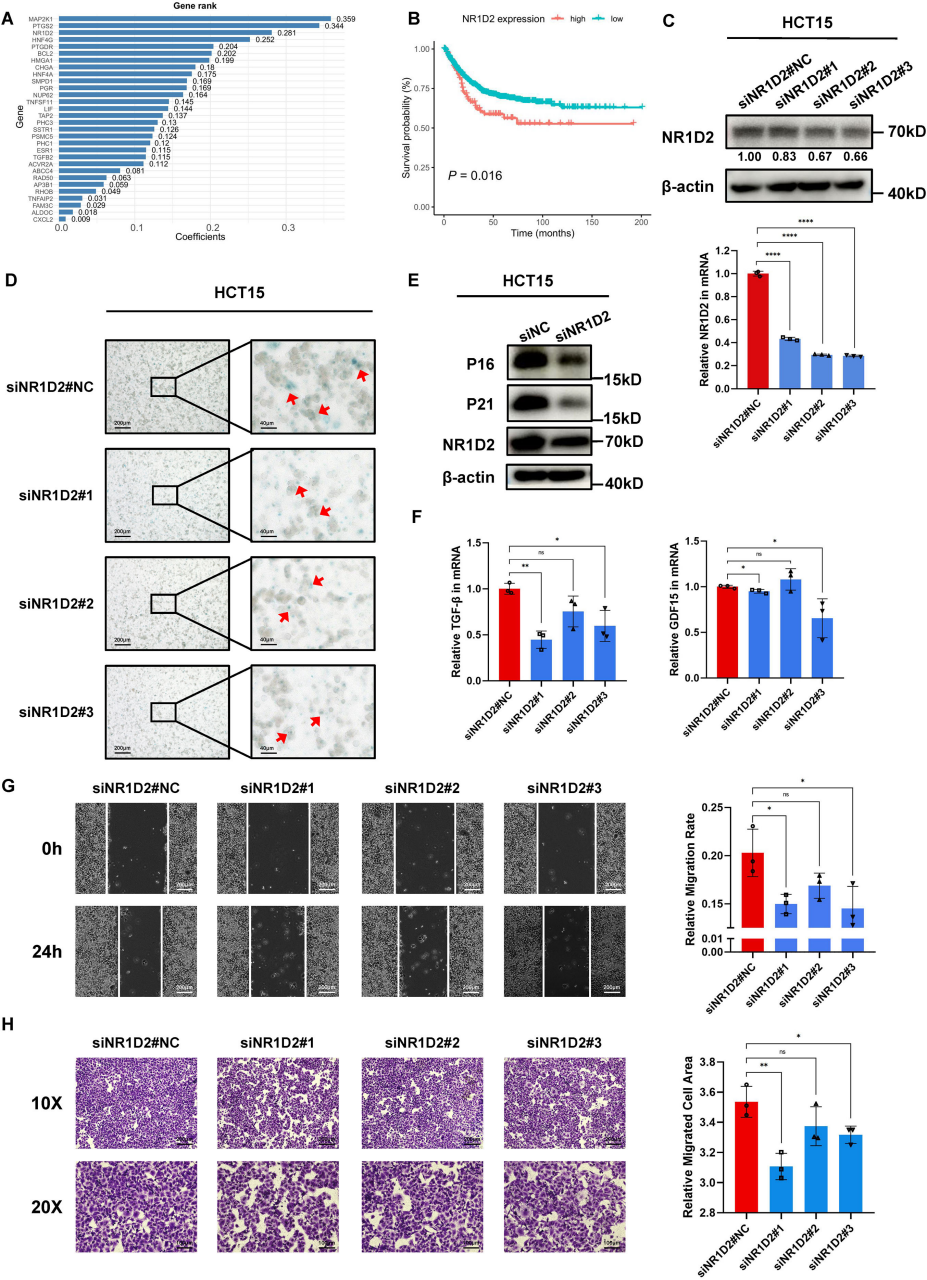
NR1D2 promotes the CRC migration. **(A)** The importance of 30 immunosenescence-related genes selected by Coxboost algorithm. **(B)** Kaplan-Meier survival curve of PFS between patients with high NR1D2 and low NR1D2 mRNA level. **(C)** The NR1D2 expression of mRNA and protein levels within native control, siNR1D2#1, siNR1D2#2 and siNR1D2#3. Relative band intensity (normalized) is shown below the WB image. **(D)** SA-β-gal staining to analyze the senescence status in the HCT15 cells within native control, siNR1D2#1, siNR1D2#2 and siNR1D2#3. **(E)** P16 and P21 protein levels assessed by Western blot after NR1D2 knocking down. **(F)** Changes in mRNA expression of SASP-associated secretory factors (TGF-β, GDF15) as determined by qRT-PCR. **(G)** Wound healing assays was performed to detect the migration within native control, siNR1D2#1, siNR1D2#2 and siNR1D2#3 in HCT15. **(H)** Transwell assays was performed to detect the migration within native control, siNR1D2#1, siNR1D2#2 and siNR1D2#3 in HCT15. * means P < 0.05, ** means P < 0.01, *** means P < 0.001, **** means P < 0.0001.

NR1D2 was identified as a pivotal gene within the MALISS signature. Stratifying patients into high- and low-expression groups based on NR1D2 mRNA levels revealed that elevated NR1D2 expression was associated with poorer PFS in stage II/III CRC patients (**Figures 4A, B**). To investigate its functional role, we knocked down (**Figure 4C**) and overexpressed (**Supplementary Figure S1A**) NR1D2 in HCT15 and confirmed the efficiency using RT−qPCR and Western blotting. Following NR1D2 knockdown, a decrease in β−galactosidase activity was observed (**Figure 4D**). The expression of P16 and P21 in protein level also indicates that NR1D2 promotes cellular senescence(**Figure 4E**, **Supplementary Figure S1B**). The senescence-associated secretory phenotype (SASP) acts as a major mediator of the paracrine effects of senescent cells in the tissue microenvironment, influencing various local and systemic biological processes, and pro-inflammatory SASP factors have been shown to promote tumorigenesis (13). RT−qPCR analysis demonstrated that NR1D2 knockdown suppressed the secretion of SASP-related factors (**Figure 4F**). To examine whether SASP factor secretion influences tumor development, we cultured normal HCT15 cells in conditioned medium from NR1D2-knockdown cells (**Supplementary Figure S1C**). Transwell and wound healing assays consistently demonstrated that NR1D2 promotes tumor migration by inducing cellular senescence and enhancing the secretion of SASP-related factors (**Figures 4G, H**, **Supplementary Figure S1D, E**).

### 3.4 The difference of gene mutational landscape among MALISS risk subgroups

**Figure 5:**
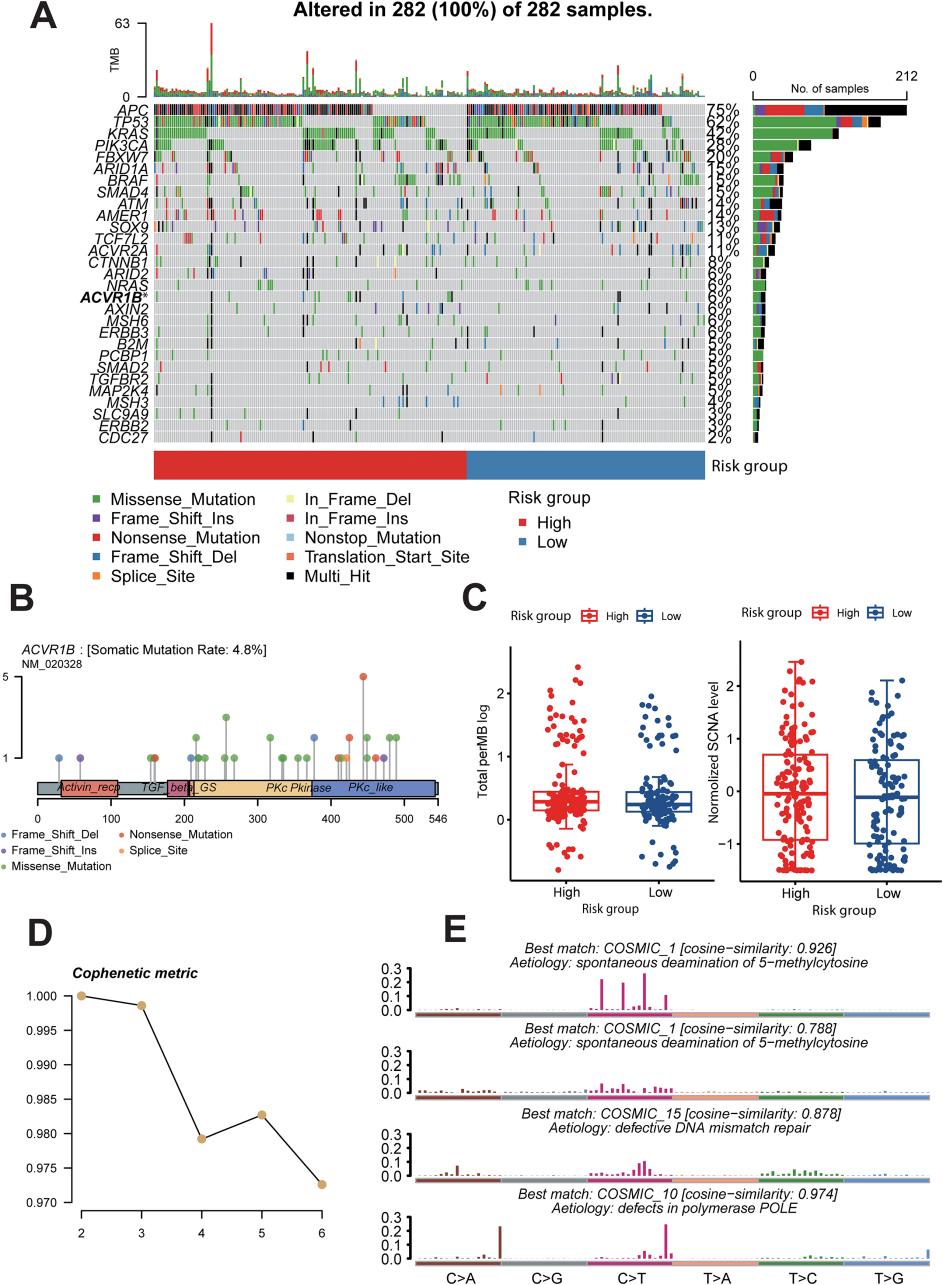
The Gene Mutational Landscape Among MALISS risk subgroups. **(A)** Mutation profile of the MALISS signature in TCGA-CRC cohorts, grouped according to low and high MALISS scores. Columns represent individual patients arranged in order of increasing MALISS score. **(B)** Distribution of somatic mutations within the ACVR1B gene represented by a lollipop diagram. **(C)** Normalized TMB and SCNA levels in high- and low-risk MALISS groups. **(D, E)** Activities of the inferred mutational signatures (Signatures 1, 15, and 10), which were matched to the COSMIC database. * means P < 0.05, ** means P < 0.01, *** means P < 0.001.

We selected gene profiles from 282 samples in a CRC database and stratified them into high-risk and low-risk groups. Based on Tumor Mutation Burden (TMB), we analyzed the types and frequencies of gene mutations in each group. In addition to common gene mutations such as APC, TP53, and KRAS mutation, we observed a notably high mutation frequency in the ACVR1B gene (P = 0.0418), which warranted further investigation (**Figure 5A**). Subsequent we analyzed the somatic mutation distribution, rate, and types of ACVR1B and revealed multiple potential mutation sites and a high mutation rate within the Protein Kinase C like (PKc-like) domain (**Figure 5B**). We also compared TMB and somatic copy number alteration (SCNA) levels between the two groups, finding that both were higher in the high-risk group, suggesting greater genomic instability in these patients (**Figure 5C**). Additionally, we extracted four mutational signatures from the genomic data with varying mutational activities and used the COSMIC database to analyze, which the tumor mutations were most likely attributed to spontaneous deamination of 5-methylcytosine, defective DNA mismatch repair, and defects in polymerase POLE (**Figures 5D, E**).

### 3.5 The tumor microenvironment associated with the MALISS

**Figure 6:**
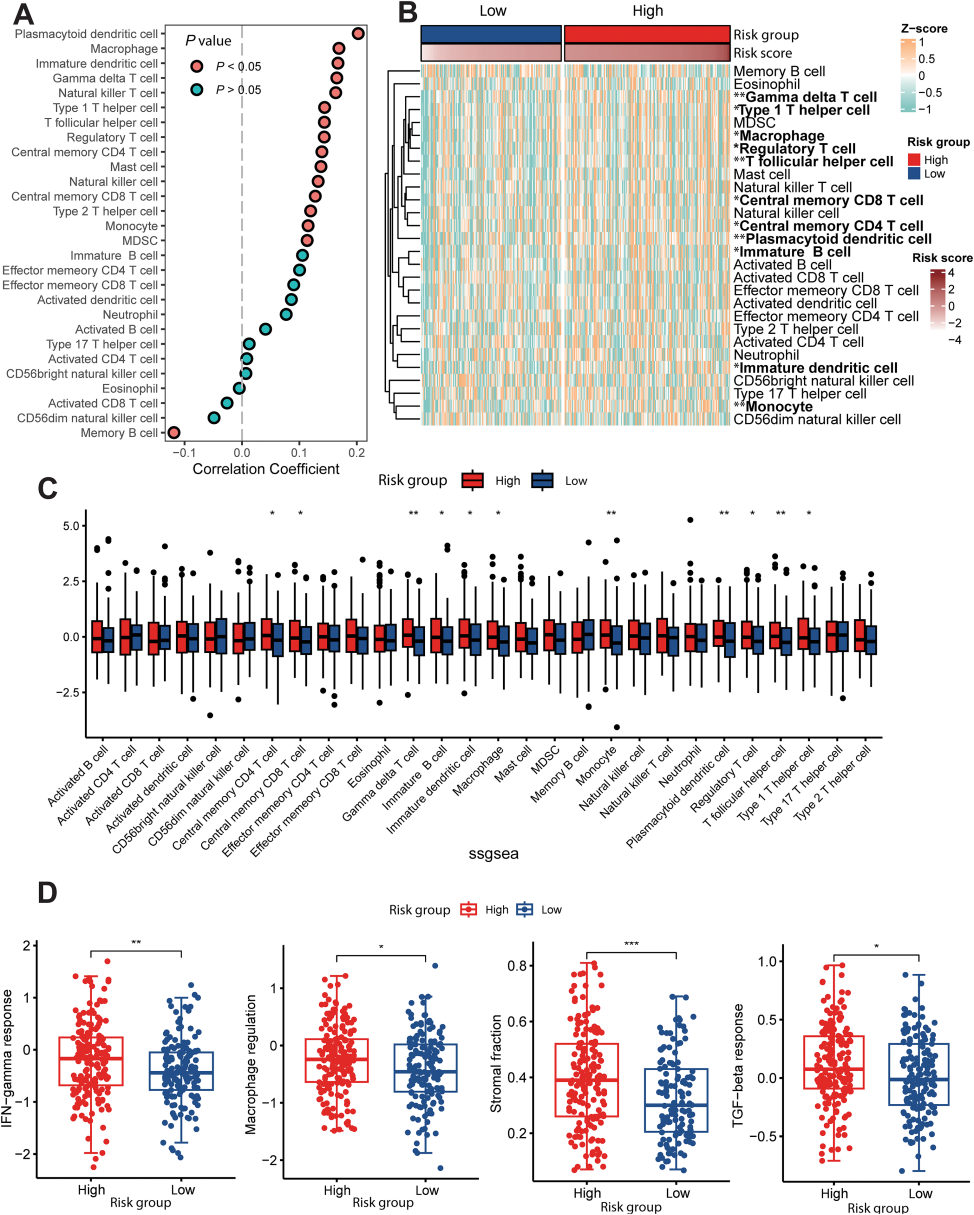
The tumor microenvironment of MALISS. **(A)** Correlation between MALISS score and immune infiltration score. **(B)** This heatmap depicts the relative abundance (Z-score) of 28 immune cell types in colorectal cancer patients, stratified into high- and low-risk groups based on the MALISS score. **(C)** The box plot illustrates the enrichment scores (Z-score) of various immune cell types in the tumor microenvironment of colorectal cancer patients, categorized into high- and low-risk groups based on the MALISS signature. Immune infiltration levels were quantified using single-sample Gene Set Enrichment Analysis (ssGSEA). **(D)** Shows the difference in IFN-γ response, Macrophage regulation, Stromal fraction, TGF-β response between the high- and low-risk groups. * means P < 0.05, ** means P < 0.01, *** means P < 0.001.

Significant cellular heterogeneity in the tumor microenvironment across samples profoundly influences tumor behavior and subsequent therapeutic decisions. First, we analyzed the correlation between the MALISS score and the immune infiltration score and found that the MALISS score may have an impact on the abundance of various immunological subsets within the tumor microenvironment. (**Figure 6A**). Then we analyzed differences in immune cell infiltration levels among MALISS subgroups and observed substantial heterogeneity across various T-cell subsets, including gamma delta T cells, type 1 T helper cells, regulatory T cells, follicular helper T cells, and central memory T cells. Additionally, significant differences were also found in other immune cells such as plasmacytoid dendritic cells and monocytes (**Figure 6B**). SsGSEA analysis of transcriptomic data from TCGA dataset further confirmed that the cell types exhibiting differential infiltration were largely consistent with those mentioned above (**Figure 6C**). Interestingly, the high-risk group showed marked enrichment of CD8^+^ T cells, Tregs and immature B cells, all of which contribute to immunosuppression within the tumor microenvironment. This may explain, the high-risk group exhibits a poorer prognosis despite substantial immune infiltration, an effect likely driven by the co-enrichment of immunosuppressive cells (14–16). The notably higher immune infiltration in the high-risk subgroup may inform subsequent immunotherapy strategies. Moreover, the high-risk group also showed elevated activity in stromal composition, IFN-γ response, TGF-β response, and macrophage regulation (**Figure 6D**).

### 3.6 Potential biological pathway concerning The MALISS

**Figure 7:**
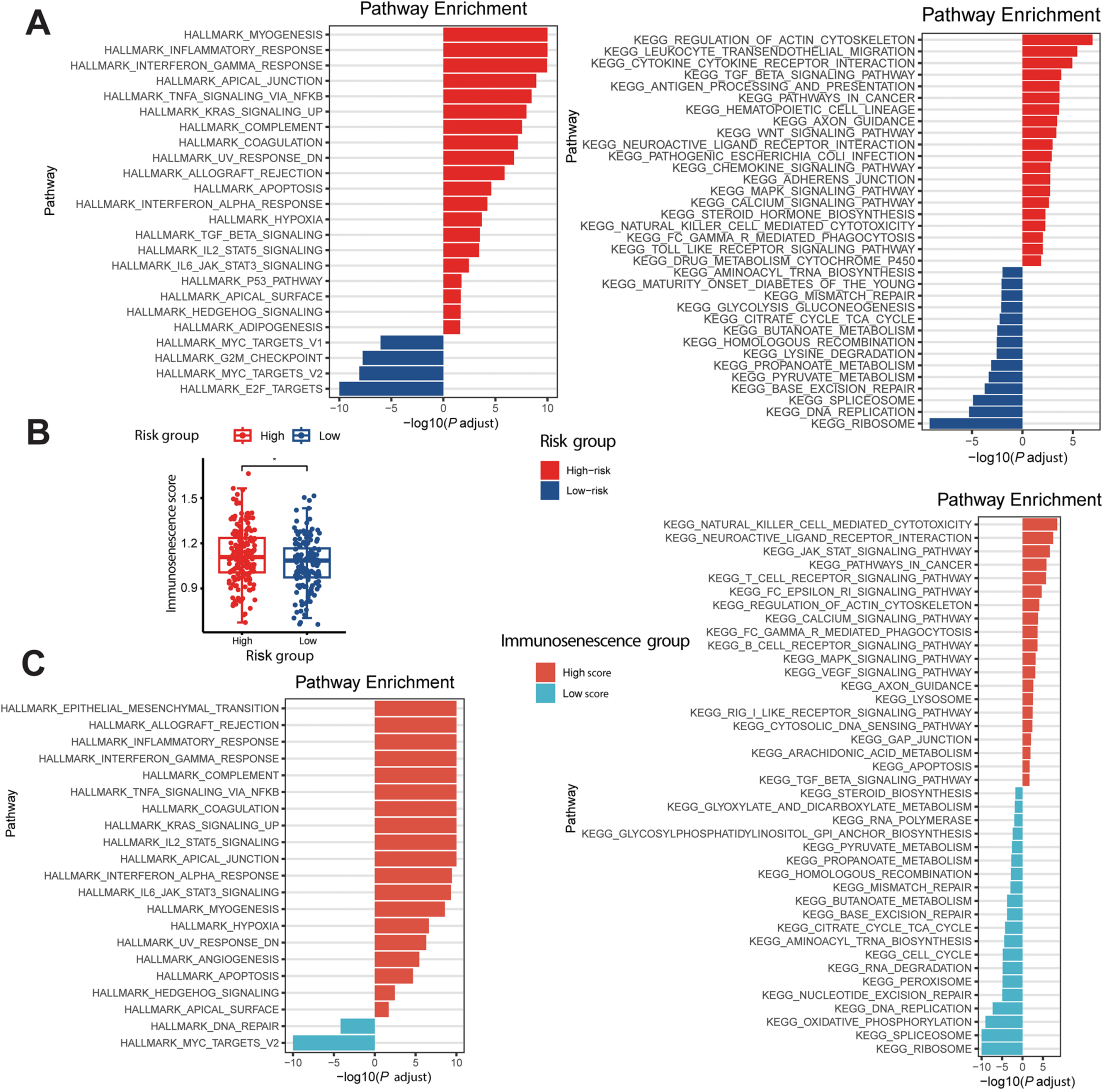
The biological pathway of MALISS. **(A)** Differential HALLMARK and KEGG Pathway Enrichment between MALISS High- and Low-Risk Groups. **(B)** Immunosenescence score between MALISS High- and Low-Risk Groups. **(C)** Differential HALLMARK and KEGG Pathway Enrichment between Immunosenescence score high and low groups. * means P < 0.05, ** means P < 0.01, *** means P < 0.001.

Using existing RNA-seq data, we performed HALLMARK and KEGG pathway analysis on different MALISS subgroups (**Figure 7A**). In both HALLMARK and KEGG database, the high-risk group showed enrichment not only in classical oncogenic signaling pathways (e.g. KRAS, JAK-STAT, and P53 signaling pathways) and tissue remodeling-related pathways (e.g. myogenesis and ACTIN cytoskeleton pathways) activated to facilitate tumor growth, but also demonstrated significant enrichment in inflammation and immune response-related pathways, including IFN-γ response and TNF-α signaling pathways. In contrast, the low-risk group was markedly enriched in E2F targets, MYC targets, DNA replication and ribosomal pathways. To further investigate the relationship between the MALISS model and immunosenescence, we assessed immunosenescence scores in both the high- and low-risk subgroups. The results revealed that the high-risk group exhibited more pronounced features of immunosenescence (**Figure 7B**). We then stratified the samples into high- and low-score groups based on immunosenescence scores and performed pathway enrichment analysis in two independent databases. Both analyses revealed pathways highly consistent with those identified in the MALISS-based stratification: the high-immunosenescence group showed enrichment in positively correlated immune-inflammatory responses and oncogenic pathway activation, while the low-immunosenescence group was enriched in negatively correlated pathways such as ribosomal biogenesis and DNA replication (**Figure 7C**).

### 3.7 MALISS model prediction in drug sensitivity

Chemotherapy is one of the primary treatment modalities in stage II/III CRC patients. To guide subsequent chemotherapy decisions, we analyzed drug sensitivity in different MALISS subgroups using the GDSC2 database. Several commonly used chemotherapeutic agents in CRC treatment, including 5-Fluorouracil, Oxaliplatin, Camptothecin, Docetaxel, and Irinotecan, demonstrated higher sensitivity in the MALISS low-risk group. In contrast, the BET inhibitor JQ1 showed greater efficacy in the high-risk group (**Supplementary Figure S2**).

### 3.8 Development and validation of the prognostic nomogram model

**Figure 8:**
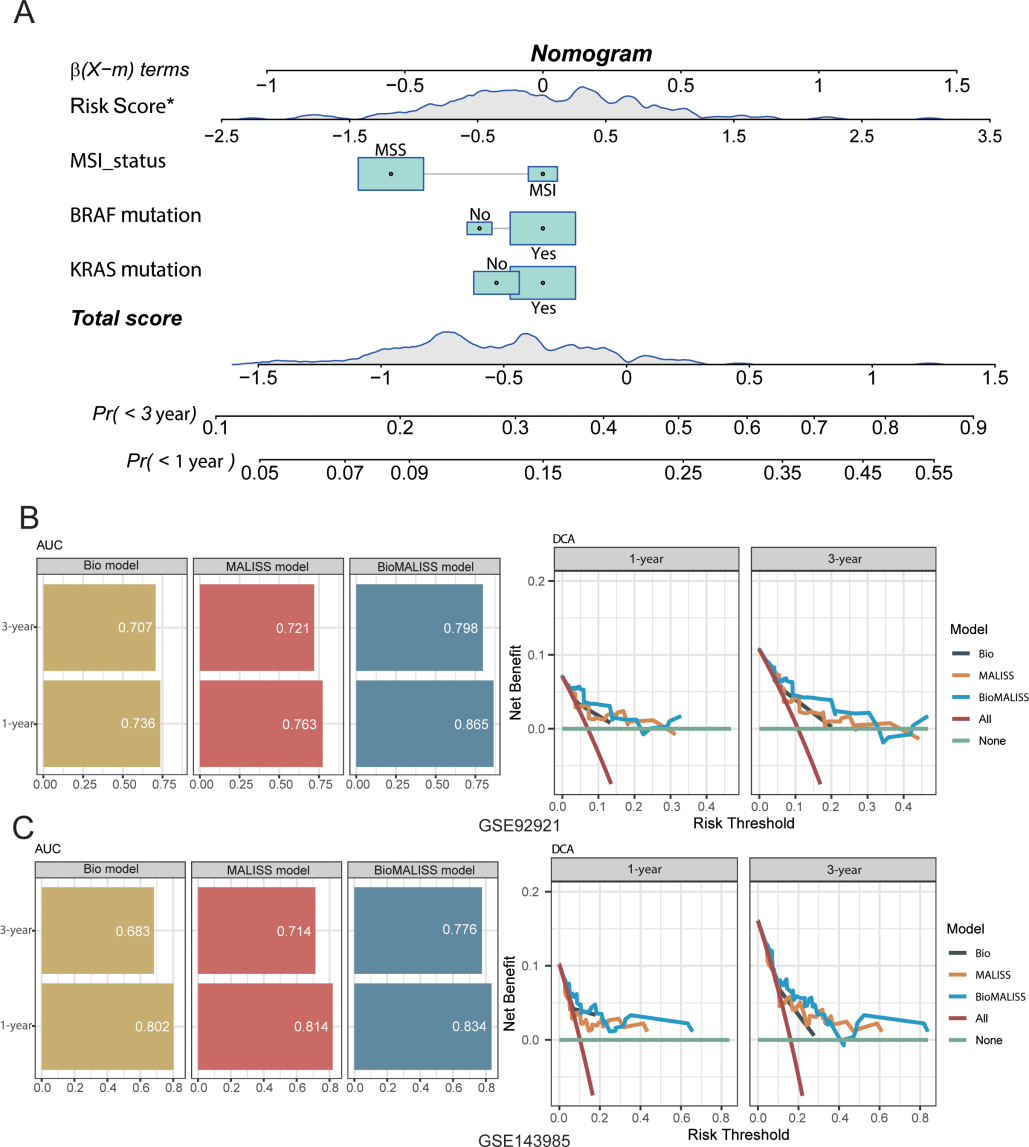
The Prognostic Nomogram Model of MALISS with molecular features. **(A)** A prognostic nomogram was developed to estimate the probability of 1−, 3−, and 5−year PFS in the TCGA dataset. **(B, C)** AUCs of Bio, MALISS and BioMALISS models for 1‐, 3‐, and 5‐year PFS probability and DCAs of Bio, MALISS and BioMALISS models for 1‐ and 3‐year PFS probability in GSE92921 and GSE143985.

To facilitate the clinical translation of the MALISS score, we integrated it with common molecular features to develop a nomogram model for predicting prognosis and recurrence risk in CRC patients. Based on biomarkers recommended by the current NCCN guidelines—including MSI status, BRAF mutation, and KRAS mutation—we combined these three indicators with the biomarker model from the training set, along with the MALISS score, to construct a nomogram named BioMALISS (**Figure 8A**). Subsequently, we performed ROC and decision curve analysis (DCA) for three different models in the GSE92921 and GSE143985 datasets (**Figures 8B, C**). The results showed that the BioMALISS model achieved significantly higher AUC values than the other two models in predicting 1-, 3-, and 5-year survival. Moreover, when the threshold probability was below 0.2, the net benefit of the BioMALISS model was notably superior to that of the other models.

## 4 Discussion

This study developed the MALISS model, which incorporates 30 immunosenescence-related prognostic genes, using a combined CoxBoost and LASSO approach. The model achieved the highest mean C-index in independent validation datasets. Unlike traditional survival analysis methods, CoxBoost is suitable for high-dimensional survival data, as it automatically selects significant variables and predicts survival time. It controls model complexity by tuning penalty parameters and the number of boosting steps, thereby avoiding overfitting (17). Meanwhile, LASSO performs feature selection in high-dimensional datasets, mitigates overfitting, and enhances both predictive accuracy and model interpretability (18). Immunosenescence represents more than just an age-related functional decline of the immune system. In the tumor microenvironment, it functions as a dysfunctional “insider,” actively fostering a pro-tumor niche that facilitates growth, immune evasion, and therapy resistance. Thus, shifting our focus from the previously established lncRNA and metabolic signatures discussed in the Introduction, we have developed a signature centered on immunosenescence—a critical and underexplored axis demanding further investigation. Among the genes included in MALISS, NR1D2 was identified as a relatively important contributor. Integrated analysis using Chronos dependency scores and prognostic association highlighted its relevance in tumor malignancy. Furthermore, *in vitro* experiments validated the role of this gene in promoting tumor cell invasion and migration. Originally known as a transcriptional repressor in circadian rhythm, the nuclear receptor NR1D2 has recently been found overexpressed in a variety of cancers (19). Furthermore, the MALISS model demonstrated a superior C-index compared to common clinical and molecular features, with favorable AUCs for 1−, 3−, and 5−year progression-free survival (PFS) across multiple datasets. These results suggest that our signature may serve as a promising biomarker for predicting high-risk recurrence in stage II/III CRC patients.

In the mutational landscape analysis of the MALISS model, we also identified evidence of selected mutations in the activin A type IB receptor (ACVR1B) gene. This finding aligns with a previous report by Kazuto Nishio et al., which demonstrated that homozygous deletion of ACVR1B in pancreatic cancer cell lines may promote an aggressive cancer phenotype by influencing SMAD2 phosphorylation and p21CIP1/WAF1 expression levels (20). Furthermore, within the MALISS high-risk subgroup, we observed a more diverse repertoire of T-cell subsets. We hypothesize that the MALISS signature and its associated mutational profile may promote cellular senescence in tumor cells, thereby remodeling the tumor immune microenvironment and ultimately fostering an immunosuppressive state. It is also worthwhile to further investigate the mechanisms underlying the development and progression of the tumor immune microenvironment in this novel CRC subtype. In drug sensitivity analysis, the BET inhibitor JQ1, which showed heightened efficacy in the high-risk subgroup, has been reported to influence tumor progression by enhancing the cytotoxicity of CD8+ T cells and reducing regulatory T cell (Treg) infiltration in the tumor microenvironment (21).

Our current model and analytical results demonstrate promising clinical applicability, though certain limitations remain. First, although a total of over 1000 patients were included in the cohort, the follow-up data are now available. Second, the specific mechanisms of action of the several immunosenescence-related genes screened from the MALISS model, as well as their precise relationships with the model itself, have yet to be fully elucidated. Third, the validity of the nomogram still requires subsequent validation in clinical cohorts and could be re-evaluated using our prospective cohort in future studies.

In summary, we established a robust and stable clinical prognostic model using a machine learning algorithm based on immunosenescence-related genes, through which numerous distinctive features were identified. Our findings demonstrate that MALISS holds significant value for predicting clinical outcomes in stage II/III CRC patients and shows promise as a practical tool for future clinical application.

## Data Availability

The datasets presented in this study can be found in online repositories. The names of the repository/repositories and accession number(s) can be found in the article/**Supplementary Material**.
