# Supplementary material for: Unveiling tumor senescence-driven prognostic heterogeneity via MALISS in stage II/III colorectal cancer
Source: Front Immunol. 2026 Jan 6;16:1744719. doi: 10.3389/fimmu.2025.1744719 (PMC12816352; doi:10.3389/fimmu.2025.1744719)
Supplement: Supplementary file 1 [file Presentation1.pdf]

Supplementary figures

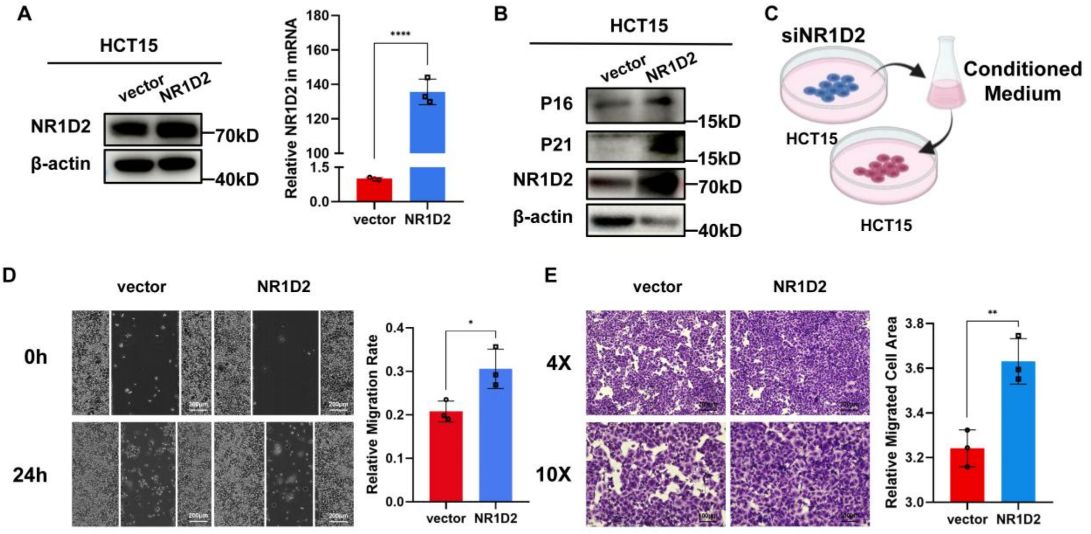

Figure S1. (A) The NR1D2 overexpression level of mRNA and protein. (B) P16 and P21 protein levels assessed by Western blot after NR1D2 overexpression. (C) Indirect co-culture model. (D-E) Wound healing assays and Transwell assays were performed to detect the migration level of NR1D2 overexpression in HCT15. * means $P < 0.05$, ** means $P < 0.01$, *** means $P < 0.001$, **** means $P < 0.0001$.

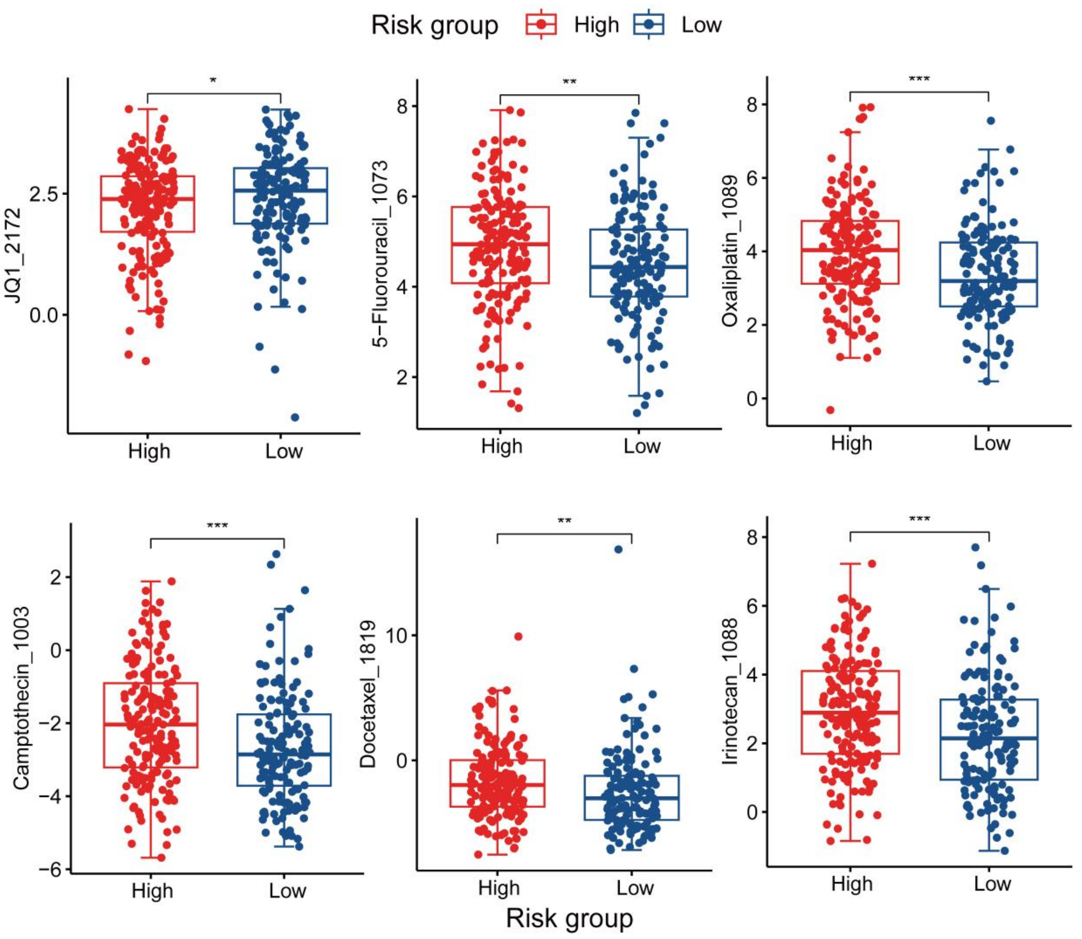

Figure S2. Comparative analysis of relative drug sensitivity between MALISS high- and low-risk groups, including JQ1, 5-Fluorouracil, Oxaliplatin, Camptothecin, Docetaxel, and Irinotecan. * means P < 0.05, ** means P < 0.01, *** means P < 0.001, **** means P < 0.0001.
